# Optimal Lymphadenectomy in Patients with Well-Differentiated Nonfunctioning Pancreatic Neuroendocrine Neoplasms

**DOI:** 10.3390/jcm12216778

**Published:** 2023-10-26

**Authors:** Ryuta Shintakuya, Kenichiro Uemura, Tatsuaki Sumiyoshi, Kenjiro Okada, Kenta Baba, Takumi Harada, Yoshiaki Murakami, Masahiro Serikawa, Yasutaka Ishii, Koji Arihiro, Shinya Takahashi

**Affiliations:** 1 Department of Surgery, Graduate School of Biomedical and Health Sciences, Hiroshima University, Hiroshima 739-8526, Japan; 2 Department of Advanced Medicine, Hiroshima University, Hiroshima 739-8526, Japan; 3 Department of Gastroenterology, Graduate School of Biomedical and Health Sciences, Hiroshima University, Hiroshima 739-8526, Japan; 4 Department of Anatomical Pathology, Hiroshima University, Hiroshima 739-8526, Japan

**Keywords:** lymph node dissection, nonfunctioning pancreatic neuroendocrine neoplasms, surgical resection

## Abstract

This study aimed to evaluate the optimal extent of lymphadenectomy in patients with nonfunctioning pancreatic neuroendocrine neoplasms. We retrospectively analyzed the clinicopathological data of patients with nonfunctioning pancreatic neuroendocrine neoplasms who underwent surgical resection. We investigated the frequency of metastases at each lymph node station according to tumor location and analyzed the factors contributing to poor overall survival (OS) and disease-free survival (DFS). Overall, data of 84 patients were analyzed. Among patients with pancreatic head tumors, metastases at stations 8, 13, and 17 were found in one (3.1%), four (12.5%), and three (9.3%) patients, respectively. However, none of the other stations showed metastases. For pancreatic body and tail tumors, metastases only at station 11 were found in two (5.1%) patients. Additionally, multivariate DFS and OS analyses showed that lymph node metastasis was the only independent prognostic factor. In conclusion, lymph node metastasis near the primary tumor was the only independent factor of poor prognosis in patients with nonfunctioning pancreatic neuroendocrine neoplasms after undergoing curative surgery. Peri-pancreatic lymphadenectomy might be recommended for nonfunctioning pancreatic neuroendocrine neoplasms.

## 1. Introduction

Approximately 2–3% of pancreatic tumors are classified as pancreatic neuroendocrine neoplasms (PNENs) [1]. Although PNENs rarely occur, their detection rate has dramatically increased over the last 3 decades owing to the proliferation of high-quality imaging techniques [1]. Based on symptoms and hormone secretion, PNENs are generally classified as functional or nonfunctional (NF-PNENs), with the majority of tumors (65–90%) being the latter [2]. The lack of early symptoms among patients with NF-PNENs often leads to late diagnosis [3]. Additionally, NF-PNENs represent the vast majority of these lesions and are characterized by various degrees of aggressiveness, including both slow-growing tumors with indolent biological behavior and aggressive neoplasms identified at an advanced stage with local invasion and/or distant metastases [4]. Previous studies reported post-curative surgery NF-PNEN recurrence rates of 13–36% [5,6,7,8]. Furthermore, several clinicopathological features, including main pancreatic duct diameter, pathological tumor size, margin status, tumor grade, perineural and microvascular invasion, and lymph node metastases (LNM), are prognostic factors after curative surgery [5,9,10,11,12,13,14]. Surgery is the mainstay of treatment for localized NF-PNENs [15,16,17]. LNM is reportedly a strong prognostic factor for patients with NF-PNENs [10,18,19]; therefore, lymph node (LN) dissection is recommended. The National Comprehensive Cancer Network (NCCN) guidelines and the Japanese Neuroendocrine Tumor Society (JNETS) recommend resection with LN dissection for tumors measuring >2 cm in size. Resection with LN dissection can also be considered for tumors measuring 1–2 cm in size [20,21]. However, only a few studies have reported the frequency of LNM at each station as well as the LN dissection degree [22,23], while the optimal lymphadenectomy extent for NF-PNENs remains controversial. Therefore, this study aimed to evaluate the optimal extent of lymphadenectomy in NF-PNENs.

## 2. Materials and Methods

### 2.1. Study Design

Samples from patients with NF-PNENs have been routinely collected since April 1993 at our institution. Therefore, the clinical data of eligible patients were collected via a retrospective review of medical records dating from April 1993 to December 2022. NF-PNENs are tumors that do not induce hormone-excess-related symptoms, which are commonly observed in PNENs [15]. Patients with functioning PNENs and multiple endocrine neoplasia and those who underwent reduction surgery for liver metastases were excluded. Asymptomatic tumors were defined as tumors that were incidentally diagnosed during clinical investigation without causing any tumor-related symptoms. Data on patients’ characteristics, surgery type, imaging findings, and pathological test results were obtained from the hospital’s electronic medical records. The main pancreatic duct diameter was measured using preoperative computed tomography (CT) from Japan. The histopathological parameters, tumor size, R status, LNM status, lymphatic invasion status, venous invasion status, perineural invasion status, Ki-67 index, and tumor grade were evaluated. Furthermore, the tumors were classified as neuroendocrine tumor (NET) grade (G)1, NET G2, NET G3, or neuroendocrine carcinoma (NEC) based on the 2017 World Health Organization (WHO) classification guidelines. NET G1 was defined as a well-differentiated tumor with a mitotic count of less than 2 per 10 high-power fields (HPF) and/or a Ki-67 index of less than 3, NET G2 as a well-differentiated tumor with a mitotic count of 2–20 per 10 HPF and/or a Ki-67 index between 3% and 20%, and NET G3 as a well-differentiated tumor with a mitotic count of more than 20 per 10 HPF and/or a Ki-67 index of greater than 20%. NEC was defined as a poorly differentiated carcinoma. This study was approved by the Ethics Review Board of Hiroshima University Hospital (identification number: E-2254), and the requirement for informed consent was waived owing to the retrospective nature of the study. All procedures were performed in accordance with the ethical standards of the 1964 Declaration of Helsinki and its later amendments, or comparable ethical standards.

### 2.2. Surgical Procedures

The operative techniques were selected according to the tumor location and size based on the preoperative imaging findings and Ki-67 index of the tumor harvested via endoscopic ultrasound-guided fine-needle aspiration. The standard surgeries performed were pancreaticoduodenectomy, distal pancreatectomy, or total pancreatectomy with regional LN dissection, and were selected according to the International Study Group on Pancreatic Surgery (ISGPS) consensus statement [24]. For some tumors measuring <10 mm in size, located at the pancreatic body or tail with a preoperative Ki-67 index of ≤3%, parenchyma-sparing resection (PSR) with LN dissection, including enucleation, spleen-preserving distal pancreatectomy (SPDP), or middle pancreatectomy (MP), was indicated. The extent of lymphadenectomy in PSR was determined according to the tumor characteristics. Surgically resected specimens were examined pathologically. The retrieved regional LNs were fixed in formalin, embedded with paraffin, sliced into 3 µm sections, and stained with hematoxylin and eosin. Two experienced pathologists determined the presence of LNM. All pathological findings were confirmed by the chief pathologist.

### 2.3. LN Stations

With regard to LNM, the location and number of harvested LNs at the LN station were recorded. The location of LNs was classified according to the ISGPS consensus statement [24]: suprapyloric LNs (station 5), infrapyloric LNs (station 6), LNs along the left gastric artery (station 7), LNs along the common hepatic artery (station 8), LNs around the celiac artery (station 9), LNs at the splenic hilum (station 10), LNs along the splenic artery (station 11), LNs along the liver hilum (station 12), LNs on the posterior aspect of the head of the pancreas (station 13), LNs along the superior mesenteric artery (station 14), LNs on the anterior surface of the head of the pancreas (station 17), and LNs along the inferior margin of the pancreas (station 18). 

### 2.4. Frequency of LNM at Each Station according to the Tumor Site

The number of patients with LNM at each station and the number of metastasized LNs in the retrieved LNs at each station were evaluated according to the tumor location. The number of patients with LNM was calculated as the number of patients with LNM at the station divided by the number of patients with LNs detected in the resected specimen at the station based on the location [22,25]. A patient was regarded as having LNM if any number of metastasized LNs was detected in the resected specimen. The number of metastasized LNs in retrieved LNs was calculated as the total number of metastasized LNs at the station divided by the total number of harvested LNs at the station based on the location [25]. 

### 2.5. Postoperative Follow-Up

All patients underwent follow-up blood tests and CT every 3–6 months. Recurrence was confirmed based on the imaging findings. The failure event for OS was defined as death from any cause. Survival time was measured from the date of surgery to the date of death or the last follow-up evaluation. DFS was calculated from the time of surgery to the time of the first radiographic evidence of recurrence or death from any cause. The recurrence patterns were classified based on the site of initial recurrence. 

### 2.6. Statistical Analysis

The associations between categorical variables were evaluated using the χ^2^ test. The Kaplan–Meier method with log-rank tests was used for performing univariate survival analysis. The factors considered significant in the univariate analysis were entered into the multivariate analysis using the Cox proportional hazards model to estimate the hazard ratios (HRs). A *p* value of <0.05 was considered significant. All statistical analyses were performed using the JMP statistical software version 15 (SAS Institute, Cary, NC, USA).

## 3. Results

### 3.1. Patient Demographics and Clinicopathological Characteristics

**Figure 1:**
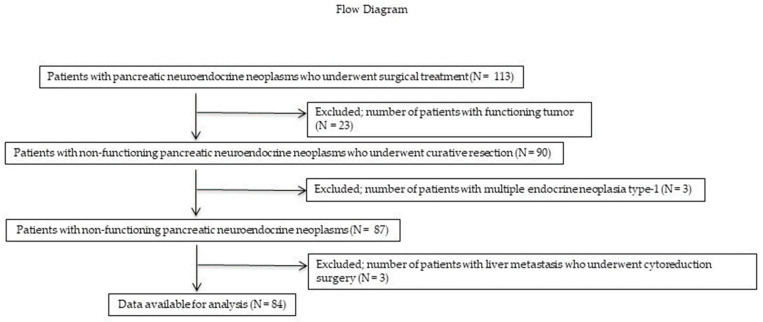
Flow diagram of the patient enrollment process. Of the 113 patients, 23 (20%) and 3 (3%) with functional PNENs and multiple endocrine neoplasia, respectively, were excluded. Three (3%) patients who underwent reduction surgery for liver metastases were also excluded. Ultimately, only 84 patients with NF-PNENs after curative surgery were eligible for inclusion in this study. PNEN, pancreatic neuroendocrine neoplasm; NF-PNEN, nonfunctioning pancreatic neuroendocrine neoplasm.

In total, 113 patients with PNENs underwent surgical resection at the Hiroshima University Hospital between January 1993 and December 2022. A flow diagram of the patient enrollment process is shown in Figure 1. Of these 113 patients, 26 (23 (0%) with functioning PNENs and 3 (3%) with multiple endocrine neoplasia) were excluded. Three (3%) patients who underwent reduction surgery for liver metastases were also excluded. Therefore, only 84 patients with NF-PNENs after curative surgery (R0 or R1 resection) were eligible for inclusion in this study. The demographic and clinicopathological characteristics of these patients are presented in Table 1. The median age was 71.2 years, and 49 (59%) patients were men. Thirty-two (38%), 20 (24%), and 32 (38%) patients had tumors in the pancreatic head, body, and tail, respectively. Fifty-nine (70%) patients underwent standard surgery with LN dissection, whereas 25 (30%) underwent PSR. Of the patients who underwent PSR, 24 underwent MP or SPDP with LN dissection. In addition, one patient underwent enucleation with station 8 LN sampling. Notably, none of the patients who underwent PSR developed LNM. Ten (12%) patients developed LNM. LNM was found in eight patients with tumors measuring >20 mm in size, and two patients with tumors measuring >15 mm and ≤20 mm; meanwhile, no LNM was found in patients with tumors measuring <15 mm. Based on the 2017 WHO classification guidelines, 48 (57%), 32 (39%), and 3 (4%) patients had NET G1, G2, and G3 tumors, respectively, whereas no patients had NEC. 

### 3.2. Frequency of LNM at Each Station according to the Tumor Site

The frequency of LNM at each LN station according to the tumor location is presented in Table 2. LNM occurred at stations 8, 13, and 17 in patients with pancreatic head tumors and at station 11 in patients with pancreatic body and tail tumors. With regard to the numbers of patients with LNM, metastases at stations 8, 13, and 17 were found in one (3.1%), four (12.5%), and three (9.3%) patients with pancreatic head tumors, respectively. However, LNM at any of the other stations was not observed. For pancreatic body and tail tumors, only metastasis at station 11 was found in two (5.1%) patients. Notably, none of the patients had LNM at any of the other stations. With regard to the numbers of metastasized LNs in retrieved LNs at each station, in patients with pancreatic head tumors, one (2.0%), seven (9.8%), and three (3.6%) metastasized LNs were found in the retrieved LNs at stations 8, 13, and 17, respectively. In patients with pancreatic body and tail tumors, three (2.2%) metastasized LNs were found in the retrieved LNs at station 11. For pancreatic head tumors, the metastases to all three stations were found in different patients.

### 3.3. Survival Analysis

**Figure 2:**
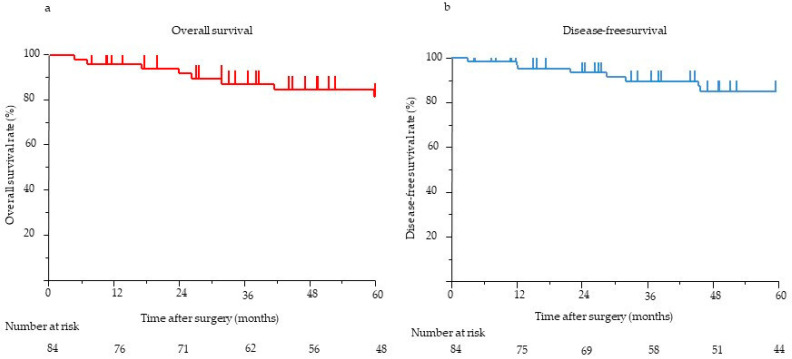
(**a**) OS of eligible patients (*n* = 84). The 5-year OS was 90.7%. (**b**) The DFS of eligible patients (*n* = 84). The 5-year DFS was 86.8%. DFS, disease-free survival; OS, overall survival.

The median postoperative follow-up time for all 84 patients was 58.1 months (range, 0.5–275.3 months). The 5-year OS and DFS rates were 90.7% and 86.8%, respectively (Figure 2). The results of the univariate and multivariate analyses of clinicopathological factors influencing DFS and OS are presented in Table 3. In the univariate analysis, pathological tumor size (>20 mm) (*p* < 0.001), margin (R1) (*p* < 0.001), LNM status (*p* < 0.001), venous invasion status (*p* < 0.001), and tumor grade (≥G2) (*p* = 0.002) were significantly associated with poor DFS. In the multivariate analysis, the presence of LNM (HR, 14.06; 95% confidence interval (CI), 1.16–169.9; *p* = 0.012) was the only independent predictor of poor DFS. In the univariate analysis, the main pancreatic duct diameter (≥5 mm) (*p* = 0.001), margin (R1) (*p* = 0.018), LNM status (*p* < 0.001), venous invasion status (*p* < 0.001), and tumor grade (≥G2) (*p* = 0.027) were significantly associated with poor OS. In the multivariate analysis, the presence of LNM (HR, 108.5; 95% CI, 1.94–6046; *p* = 0.005) was the only independent predictor of poor OS. Eight (9%) patients showed recurrence on imaging. Recurrence frequently occurred in the liver (*n* = 7), followed by the peritoneum (*n* = 1). None of the patients experienced local recurrence. Six patients with LNM experienced recurrence: one in the peritoneum and five in the liver. All eight patients with recurrence had G2 or G3 disease, although the tumor grade (≥G2) was not independently associated with poor DFS. Further, we considered risk factors for the most frequent recurrence, which was recurrence in the liver. In the univariate analysis, pathological tumor size (>20 mm) (*p* = 0.002), the presence of LNM (*p* < 0.001), the presence of venous invasion (*p* < 0.001), and tumor grade (≥G2) (*p* = 0.002) were significantly associated with recurrence in the liver. In the multivariate analysis, the presence of LNM (odds ratio, 17.8; 95% CI, 1.20–263.97; *p* = 0.036) was the only independent risk factor for recurrence in the liver.

## 4. Discussion

The NCCN and JNETS guidelines recommend resection with LN dissection for patients with tumors measuring >2 cm and resection with LN dissection for tumors measuring 1–2 cm [20,21]. Nevertheless, the optimal extent of lymphadenectomy in NF-PNENs based on tumor location nevertheless remains unclear. In the current study, metastases at stations 8, 13, and 17 were found in patients with pancreatic head tumors, and none of the patients had LNM at other stations. In patients with pancreatic body and tail tumors, metastasis only at station 11 was found. A few recent studies have reported the frequency of LNM to each station and found metastasis at station 14 in addition to stations near the primary tumor in patients with resected NF-PNENs [22,23]. However, the current study reported the absence of metastasis at station 14. Masui et al. [23] reported the frequency of LNM to each station according to the tumor location in patients with resected NF-PNENs with tumors measuring ≤20 mm in size; moreover, metastases were also found at station 14 in addition to the stations near the primary tumor that developed in the pancreatic uncus and body. Takagi et al. [22] reported the prognostic value of the regional LN station according to the tumor location in patients with resected NF-PNENs; they found metastases at stations 8, 13, 14, and 17 among patients with pancreatic head tumors and metastasis at station 11 in those with pancreatic body or tail tumors. In the current study, the frequency of LNM was lower in pancreatic body or tail tumors than in pancreatic head tumors. LNM occurred only in 2 (5.1%) patients with pancreatic body or tail tumors. In contrast, LNM occurred in 8 (20.0%) patients with pancreatic body or tail tumors. The frequency of LNM in patients with pancreatic body or tail tumors was similar to that reported in a previous study by Takagi et al. [22]; they reported that LNM occurred in 2 (6.7%) of the 30 patients with pancreatic body or tail tumor and in 7 (35%) of the 20 patients with pancreatic head tumors.

Our results identified LNM as a predictor of poor prognosis in PNENs, concurring with the findings of previous studies [10,18,19,26]. Tan et al. [19] investigated the predictors of recurrence in patients with well-differentiated NF-PNENs after curative surgery and reported that LNM is most strongly associated with recurrence, and that recurrence is seven times more frequent in patients with LNM than in those without LNM [19]. In terms of the predictors of poor OS, Fischer et al. [18] reported that tumor grade G3, the presence of distant metastasis, and the presence of LNM were independent predictors of poor OS in patients with PNENs after surgical resection [18]. Partelli et al. investigated the predictors of recurrence, including the examined LN, LN ratio, and number of LNM (N0; N1: 1–3 positive LN; N2: >3 positive LN) in 157 patients with well-differentiated NF-PNEN after curative surgery. They found that the presence of tumor necrosis, LN ratio (>0.40), and the number of LNM (N1 or N2) were independent predictors of a poor DFS [27]. Tumor size, margin, tumor grade, perineural invasion status, and microvascular invasion status are poor prognostic factors [5,11,12,13,14,28] but were not associated with poor prognosis in our study. Therefore, the prognostic value of these factors remains unclear [11]. 

In previous studies, the recurrence rates of patients with NF-PNENs after curative surgery were reportedly 13–36% [5,6,7,8]. The recurrence rate in this study was 10%, which was relatively lower than that in previous reports. Although recurrence in the liver is considered the most frequent type of recurrence [6,19,29], no studies have investigated the risk factors for recurrence in the liver in patients with NF-PNENs. In this study, only LNM was independently associated with recurrence in the liver. LNM might therefore be a systemic factor because recurrence in the liver has a hematogenous spread. Additionally, no previous study has evaluated the effect of adjuvant treatment in patients with well-differentiated NF-PNENs after curative surgery. The NANETS, NCCN, and JNETS NET guidelines currently do not recommend systemic treatment for patients with well-differentiated NF-PNENs after curative surgery [20,21,30]. However, some systemic treatments might be required for patients with well-differentiated NF-PNENs who exhibit high-risk factors for systemic recurrences, such as LNM, to control occult micrometastasis and prevent distant metastasis. 

This study has some limitations. First, this was a single-center retrospective study with a relatively small sample size owing to the rarity of the disease; thus, bias was unavoidable. Second, the optimal extent of lymphadenectomy might be different for body and tail tumors; therefore, a separate evaluation of the frequency of LNM at each station in patients with body and tail tumors is required. In this study, only the frequency of LNM in patients with body and tail tumors was evaluated owing to the small number of patients with LNM at each station. Third, the frequency of LNM in patients with pancreatic body and tail tumors might have been overestimated because the extent of lymphadenectomy in PSR, such as MP, SPDP, and enucleation, was determined according to the tumor characteristics. In fact, LNMs at stations 10, 11, and 18 in the resected specimen were detected in 29, 39, and 37 patients, respectively, although a total of 52 patients had pancreatic body or tail tumors. Fourth, LNMs were found only in 2 (5.1%) patients with pancreatic body or tail tumors; therefore, significant conclusions cannot be drawn from such limited data, and multicenter studies with a larger sample size are required to validate our findings.

## 5. Conclusions

In patients with pancreatic head tumors, LNM were found at stations 8, 13, and 17. In contrast, in patients with pancreatic body and tail tumors, LNM were found only at station 11. None of the patients had LNM at station 14. LNM was the only independent factor of poor prognosis in patients with NF-PNENs after curative surgery. Thus, peri-pancreatic lymphadenectomy might prolong the survival of patients with NF-PNENs. However, future multicenter studies with a larger sample size are required to further elucidate the optimal extent of lymphadenectomy in patients with NF-PNENs.

## Figures and Tables

**Table 1:** Demographic data, clinicopathological characteristics, and pathological factors of patients with NF-PNENs who underwent curative surgery (*n* = 84).

Variable	
Age, median (IQR), years	71.2 (39–82)
Sex	
Male/female	49 (58%)/35 (42%)
BMI, median (IQR), kg/m^2^	22.8 (15.7–32.6)
Tumor location	
Head/body/tail	32 (38%)/20 (24%)/32 (38%)
Procedure	
Standard surgery	59 (71%)
PD/DP/TP	32 (38%)/27 (33%)/0 (0%)
PSR	25 (29%)
MP/SPDP/enucleation	12 (14%)/12 (14%)/1 (1%)
Main pancreatic duct, median (IQR), mm	2.0 (1–13)
Pathological tumor diameter, median (IQR), mm	10.0 (3–90)
R0 rate	
R0/R1/R2	79 (94%)/5 (6%)/0 (0%)
Lymph node metastasis	
Yes/No	10 (12%)/74 (88%)
Lymphatic invasion	
Yes/No	11 (14%)/66 (86%)
Venous invasion	
Yes/No	19 (25%)/58 (75%)
Perineural invasion	
Yes/No	17 (20%)/66 (80%)
Ki-67 (%), median (IQR)	1.3 (0–50)
WHO classification	
G1/G2/G3	48 (57%)/32 (38%)/3 (5%)
Follow-up, median (IQR), months	58.1 (range, 0.5–275.3)

BMI, body mass index; DP, distal pancreatectomy; IQR, interquartile range; MP, middle pancreatectomy; NF-PNENs, nonfunctioning pancreatic neuroendocrine neoplasms; PD, pancreaticoduodenectomy; PSR, parenchyma-sparing resection; SPDP, spleen-preserving distal pancreatectomy; TP, total pancreatectomy; WHO, World Health Organization.

**Table 2:** Frequency of LNM at each station according to the tumor site.

	Pancreatic Head (*n* = 32)		Pancreatic Body and Tail (*n* = 52)	
	Number of Patients with LNM/Number of Patients with LNs Detected in the Resected Specimen	Total Number of Metastasized LNs/Total Number of Harvested LNs	Number of Patients with LNM/Number of Patients with LNs Detected in the Resected Specimen	Total Number of Metastasized LNs/total Number of Harvested LNs
Station 5	0/26 (0%)	0/38 (0%)	NA	NA
Station 6	0/30 (0%)	0/42 (0%)	NA	NA
Station 7	0/4 (0%)	0/7 (0%)	0/29 (0%)	0/39 (0%)
Station 8	1/32 (3.1%)	1/51 (2.0%)	0/37 (0%)	0/52 (0%)
Station 9	0/2 (0%)	0/4 (0%)	0/19 (0%)	0/28 (0%)
Station 10	NA	NA	0/29 (0%)	0/44 (0%)
Station 11	NA	NA	2/39 (5.1%)	3/132 (2.2%)
Station 12	0/27 (0%)	0/38 (0%)	NA	NA
Station 13	4/32 (12.5%)	7/71 (9.8%)	NA	NA
Station 14	0/30 (0%)	0/72 (0%)	0/23 (0%)	0/36 (0%)
Station 17	3/32 (9.3%)	3/82 (3.6%)	NA	NA
Station 18	NA	NA	0/37 (0%)	0/52 (0%)

LNM, lymph node metastasis; LN, lymph node; NA, not available.

**Table 3:** Univariate and multivariate survival analyses of the clinicopathological factors of all 84 patients.

Factor		DFS						OS					
		Univariate Analysis			Multivariate Analysis			Univariate Analysis			**Multivariate Analysis**		
	No.	Five-Year Survival Rate (%)	Median DFS (Months)	*p* Value	HR	95% CI	*p* Value	Five-Year Survival Rate (%)	Median OS (Months)	*p* Value	**HR**	**95% CI**	*p* Value
Sex													
Male	49 (58%)	88.2	NA	0.805				86.0	NA	0.657			
Female	35 (42%)	87.1	NA					90.9	NA				
Tumor location													
Head	32 (38%)	85.7	NA	0.884				76.3	NA	0.183			
Body, tail	52 (62%)	87.2	NA					94.5	NA				
Main pancreatic duct diameter													
≥5 mm	13 (15%)	77.8	NA	0.115				63.5	142.6	0.001	6.34	0.34–111.5	0.192
<5 mm	70 (85%)	89.6	NA					95.7	NA		1.0		
Pathological tumor size													
>20 mm	19 (23%)	67.3	NA	<0.001	1.32	0.074–23.64	0.848	85.3	142.6	0.481			
≤20 mm	65 (77%)	97.8	NA		1.0			91.2	NA				
R1													
Yes	5 (6%)	30.1	32.2	<0.001	2.67	0.44–16.2	0.283	80.0	113.4	0.018	16.3	0.0015–2.48	0.104
No	79 (94%)	91.4	NA		1.0			88.1	NA		1.0		
Lymph node metastasis													
Yes	10 (12%)	26.7	32.2	<0.001	14.06	1.16–169.9	0.012	76.1	87.57	<0.001	108.5	1.94–6046.2	0.005
No	74 (88%)	96.1	NA		1.0			88.9	NA		1.0		
Lymphatic invasion													
Yes	11 (14%)	78.5	NA	0.312				90.0	NA	0.208			
No	66 (86%)	86.0	NA					95.7	NA				
Venous invasion													
Yes	19 (25%)	51.6	NA	<0.001	3.1	0.21–44.8	0.357	87.4	113.4	<0.001	1.01	0.019–51.74	0.995
No	58 (75%)	97.9	NA		1.0			97.4	NA		1.0		
Perineural invasion													
Yes	17 (20%)	83.0	NA	0.704				85.5	NA	0.952			
No	66 (80%)	87.6	NA					87.9	NA				
WHO classification of specimens													
G1	48 (58%)	72.3	NA	0.002	1.0			86.4	NA	0.027	1.0		
G2, G3	35 (42%)	50.0	NA		8.04	0.83–77.9	0.076	60.2	NA		3.71	0.043–313.0	0.560

CI, confidence interval; DFS, disease-free survival; HR, hazard ratio; OS, overall survival; NA, not available; WHO, World Health Organization.

## Data Availability

The data presented in this study are available from the corresponding author upon reasonable request. The data are not publicly available due to privacy issues.
